# A Subpopulation of Schwann Cell-Like Cells With Nerve Regeneration Signatures Is Identified Through Single-Cell RNA Sequencing

**DOI:** 10.3389/fphys.2021.637924

**Published:** 2021-05-10

**Authors:** Zairong Wei, Shenyou Shu, Mingjun Zhang, Sitian Xie, Shijie Tang, Kaiyu Nie, Haihong Li

**Affiliations:** ^1^Department of Plastic Surgery and Burn Center, The Second Affiliated Hospital, Shantou University Medical College, Shantou, China; ^2^Department of Wound Repair and Dermatologic Surgery, Taihe Hospital, Hubei University of Medicine, Shiyan, China

**Keywords:** stem cells, Schwann cell-like cells, single-cell RNA sequencing, nerve regeneration, NRG1

## Abstract

Schwann cell-like cells (SCLCs) derived from human amniotic mesenchymal stem cells (hAMSCs) have been shown to promote peripheral nerve regeneration, but the underlying molecular mechanism was still poorly understood. In order to investigate the heterogeneity and potential molecular mechanism of SCLCs in the treatment of peripheral nerve regeneration at a single cell level, single-cell RNA sequencing was applied to profile single cell populations of hAMSCs and SCLCs. We profiled 6,008 and 5,140 single cells from hAMSCs and SCLCs, respectively. Based on bioinformatics analysis, pathways associated with proliferation, ECM organization, and tissue repair were enriched within both populations. Cell cycle analysis indicated that single cells within these two populations remained mostly in the G0/G1 phase. The transformation of single cells from hAMSCs to SCLCs was characterized by pseudotime analysis. Furthermore, we identified a subpopulation of SCLCs that highly expressed genes associated with Schwann cell proliferation, migration, and survival, such as JUN, JUND, and NRG1., Genes such as PTGS2, PITX1, VEGFA, and FGF2 that promote nerve regeneration were also highly expressed in single cells within this subpopulation, and terms associated with inflammatory and tissue repair were enriched in this subpopulation by pathway enrichment analysis. Our results indicate that a subpopulation of SCLCs with nerve regeneration signatures may be the key populations that promote nerve regeneration.

## Introduction

Peripheral nerve injury (PNI) is widely encountered in clinical practice. Its incidence is about 0.18% in developed countries, with this percentage being higher in developing countries (Modrak et al., 2019). Following a PNI, the damaged nerve can be regenerated through nerve end-to-end anastomosis, autologous nerve transplantation, or nerve conduits (Kubiak et al., 2020). However, the nerve regeneration outcomes of these treatments, and especially the recovery of nerve function, are not always favorable, with painful neuroma, anastomotic scars, and flawed development of motor sensory nerve axons being commonly reported (Sullivan et al., 2016; Han et al., 2019).

The ability of peripheral nerves to regenerate autonomously after an injury is mainly contingent on the actions of inherent supporting cells such as Schwann cells (Jessen et al., 2015; Bolívar et al., 2020; Nocera and Jacob, 2020). As the primary glia and myelin-forming cells within the peripheral nervous system, Schwann cells play an important role in nerve regeneration (Jessen et al., 2015; Bolívar et al., 2020; Nocera and Jacob, 2020), initiating a reprogramming process after PNI in which they demyelinate and transdifferentiate into repair Schwann cells (Nocera and Jacob, 2020). Several transcription factors and pathways support Schwann cell proliferation, migration, and survival during this process (Monje, 2020; Nocera and Jacob, 2020). Notable among these factors are JUN, SOX2, and STAT3, which play a critical role in Schwann cell reprogramming (Nocera and Jacob, 2020). As a key regulator of Schwann cells, JUN is involved throughout the PNI repair process (Arthur-Farraj et al., 2012; Jessen et al., 2015). NRG1/ErbB (Newbern and Birchmeier, 2010), MAPK (Ishii et al., 2014), and Notch (Woodhoo et al., 2009) have been identified as key signaling pathways that support the survival, proliferation, and migration of Schwann cells. Apart from transcription factors and pathways, secreted proteins, such as VEGFA, NGF, BDNF, and GNDF (Scheib and Höke, 2013) also play an important role in Schwann cell reprogramming (Scheib and Höke, 2013). However, given a lack of donors and poor *in vitro* proliferation, the clinical application of Schwann cells remains limited. The issue of how Schwann cells can be cultured in sufficient quantities *in vitro*, while maintaining their functions is a challenging one.

In recent years, stem cells have been found to have a promising role in tissue repair and organ reconstruction. Stem cell-based cell therapy has gained prominence within clinical practice (Rameshwar et al., 2018; Kubiak et al., 2020). There are three methods of applying stem cell transplantation in PNI repair: differentiation into Schwann cell-like cells (SCLCs) (Choi et al., 2021), increasing the expression of neurotrophic factors, and promoting the myelination of regenerated axons (Jiang et al., 2017; Hopf et al., 2020; Yi et al., 2020; Lavorato et al., 2021). Stem cells induced using several compounds can subsequently be differentiated into SCLCs. Not only stem cells, Schwann cells can also be induced from adult human fibroblasts (Thoma et al., 2014; Sowa et al., 2017; Kitada et al., 2019; Kim et al., 2020). Surface markers of Schwann cells, such as GFAP, p75, and S100, are highly expressed in SCLCs (Dore et al., 2009). SCLCs can accelerate the regeneration of transected axons and myelin sheaths more expeditiously than Schwann cells alone (Wang et al., 2011; Tomita et al., 2012; Hopf et al., 2020). Moreover, they create a suitable microenvironment for the growth of Schwann cells, which secrete more neurotrophic factors and accelerate the formation of myelin sheaths around regenerated axons (Jiang et al., 2017). Previously, we reported that SCLCs derived from human amniotic mesenchymal stem cells (hAMSCs) promote sciatic nerve regeneration by activating the miR214/c-JUN pathway (Chen et al., 2019). However, the heterogeneity of SCLCs remains poorly understood.

Applying single-cell RNA sequencing technology (scRNA-seq), we investigated the heterogeneity of single cells in hAMSCs and SCLCs. We further performed cell cycle and Pseudotime analysis to investigate cell distribution during cell cycle phases and traced the process of transformation of hAMSCs into SCLCs. We identified and profiled a subpopulation of SCLCs that highly expressed Schwann cell marker genes.

## Materials and Methods

### Extraction of hAMSCs and Cell Culture

As described in a previous study (Dezawa et al., 2001; Chen et al., 2019), we extracted and developed a cell culture of hAMSCs. Briefly, the amniotic membrane was stripped under aseptic conditions from different patients’ abandoned placenta. After being rinsed and cleaned with phosphate-buffered saline (PBS, Solarbio, P1010) containing 1% penicillin and streptomycin solution (Solarbio, P1400), the amniotic membrane was cut into pieces and digested within a solution of 0.05% trypsin (Solarbio, T1300) for a period of 1 h. The digested mixture was then passed through a 300-mesh filter sieve, and any undigested tissue was completely digested by collagenase type II (0.5 mg/mL, Solarbio, C8150). The mixture was filtered and centrifuged, and the cells were suspended in DMEM/F12 (Gibco, 31330095) complete medium containing 10% Fetal Bovine Serum, (FBS, Gibco, 12664025), 1% L-glutamine (Solarbio, G8230), 1% penicillin and streptomycin (Solarbio, P1400), and 10ng/mL basic fibroblast growth factor, (bFGF, peprotech, 100-18B) and incubated at 37 °C with 5% CO_2_. hAMSCs were characterized by immunofluorescence and flow cytometry as previously described before (Chen et al., 2019) (Supplementary Figure 1).

### Induction of hAMSCs Into SCLCs

To differentiate hAMSCs (passage 2) into SCLCs, we first cultured them for 24 h in DMEM-F12 (Gibco, 31330095) complete medium containing 1 mmol/L Mercaptoethanol, (Solarbio, M8210) at 37°C with 5% CO_2_. The medium was then replaced with DMEM-F12 (Gibco, 31330095) complete medium containing 35 ng/mL All-trans-retinoic acid (ATRA, Sigma, R2625) and cultured for 72 h. Lastly, the cells were cultured with DMEM-F12 (Gibco, 31330095) complete medium containing 5 mmol/L forskolin (Sigma, F3917), 10 ng/ml bFGF (peprotech, 100-18B), 5 ng/ml Platelet Derived Growth Factor-AA, (PDGF-AA, peprotech, 100-13A), and 200 ng/ml Heregulinβ-1, (peprotech, 100-03) for a 2-week period.

### Total RNA Isolation and Sequencing

We used TRIzol (Invitrogen, 15596018)) to extract the total RNA of hAMSCs and SCLCs, following the manufacturer’s instructions. Three samples for each group RNA electrophoresis, Qubit (Thermo Fisher) and the Agilent 2100 Bioanalyzer system were used to measure the concentration and quality of the total RNA. Illumina sequencing libraries were constructed by Novogene Co., Ltd., which also performed pair-end sequencing with a read length of 2 × 150 bp (Illumina HiSeq3000).

### Bulk RNA-Seq Analysis

After performing data filtering and quality control of the raw RNA-seq data, we aligned the filtered reads to the human genome (GENCODE GRCh38) using the STAR software (Dobin et al., 2013). Differentially-expressed genes (DEGs) were identified using the R package, DESeq2 (Love et al., 2014); genes with Log2 (fold change) ≥ 1 and a p-value ≤ 0.05 were defined as DEGs. A volcano plot of DEGs was generated using the following R packages: ggthemes (Arnold et al., 2019) and ggpubr (Kassambara, 2020).

### Single-Cell Sample Preparation and RNA Sequencing

The cultured hAMSCs and SCLCs were first digested in a 0.05% trypsin (Solarbio, T1300) solution and then re-suspended in pre-cooled PBS (Solarbio, P1010) containing 0.04% BSA (Solarbio, A8010). One sample for each group. We prepared a single-cell library, following the manufacturer’s instructions (Chromium Single Cell 3′ v2 Reagent Kit; 10X Genomics, Pleasanton, CA, United States). Briefly, the cell suspension (2,000 cells/μl) was loaded on to Chromium microfluidic chips. The mRNA from the barcoded cells was subsequently reverse-transcribed, and sequencing libraries were constructed using reagents from the kit according to the manufacturer’s instructions. Sequencing (NovaSeq) was performed by Novogene. RNA-seq data used in this study were deposited into the GEO database (GSE161066).

### Single-Cell RNA-Seq Data Analysis

Reads generated from raw scRNA-seq data were demultiplexed and mapped to the human genome (GENCODE GRCh38) using the STAR software (Dobin et al., 2013) in the 10X Genomics CellRanger pipeline with default parameters. All downstream analysis was performed using the CellRanger software and Seurat toolkit (Macosko et al., 2015; Powers and Satija, 2015; Butler et al., 2018). In brief, the single-cell data were initially filtered using CellRanger with default parameters, and unique molecule identifiers (UMI) were then counted to construct digital expression matrices. The single-cell data were subsequently filtered using Seurat according to the following principles: a gene that was expressed in more than three cells was considered expressed, and any cell with at least 200 expressed genes was deemed a single cell. Seurat was used to perform data normalization, dimensionality reduction, clustering, differential expression, and cell-cycle scoring (Powers and Satija, 2015; Butler et al., 2018).

### Pseudotime Analysis

Single-cell trajectory analysis was performed using the R package, Monocle (Qiu et al., 2017). First, a Cell DataSet matrix was created for single cells using the default parameters. Next, we followed two major steps to construct the single-cell trajectory. During the first step, we chose genes with important information that could be used to define the progression of cell transition. The second step entailed dimensionality reduction and trajectory construction with the ordering genes. A reversed graph embedding algorithm was applied by projecting cells on to a low dimensional space, while simultaneously learning smooth treelike manifold. We constructed separate single-cell trajectories of hAMSCs, SCLCs, and hAMSCs plus SCLCs.

### Functional Enrichment Analysis

We performed Gene Ontology (GO) and Kyoto Encyclopedia of Genes and Genomes (KEGG) functional enrichment analyses of differentially expressed genes using the R package, ClusterProfiler (Yu et al., 2012). GO terms and KEGG pathways with corrected p-values of less than 0.05 were considered significantly enriched. Venn diagrams in this manuscript were generated using tools sourced from http://bioinfogp.cnb.csic.es/tools/venny/. Bar plots and pie charts were generated using GraphPad Prism 7.

### Statistical Analysis

To identify differentially expressed genes in bulk RNA-seq data or scRNA-seq, DESeq2 and Seurat were used to analysis the expression data of bulk RNA-seq and scRNA-seq, respectively. Genes with Log2 (Fold change) ≥ 1 and *p* value ≤ 0.05 were defined as differentially expressed. Data were shown as mean ± standard deviation or mean ± standard error of mean (SEM), as indicated. *P* value < 0.05 was considered as significance at the 95% confidence level.

## Results

### Overview of the scRNA-Seq Data Generated From hAMSCs and SCLCs

**FIGURE 1:**
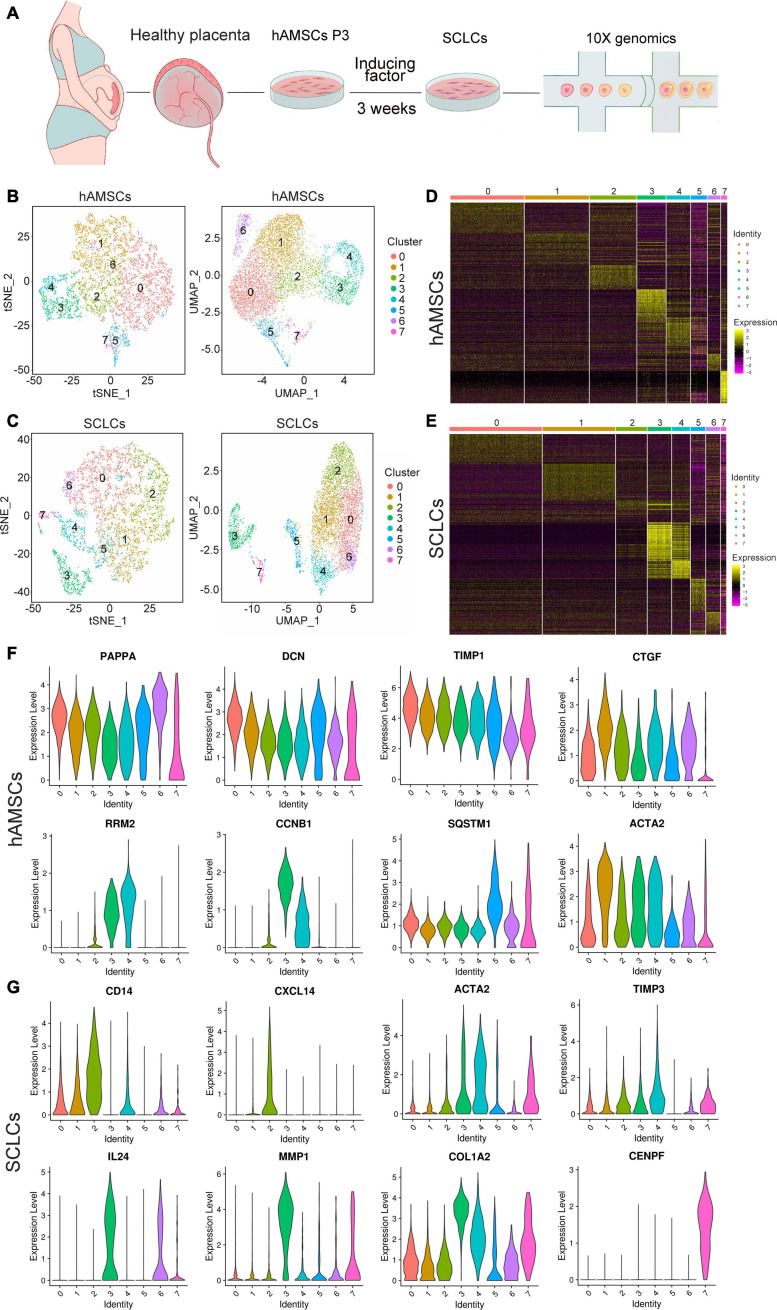
Overview of scRNA-seq data generated from hAMSCs and SCLCs. **(A)** Schematic depiction of the overall workflow. **(B)** tSNE or UMAP plots of 6,008 cells generated from hAMSCs. The plots are colored by cell cluster, and the cells are clustered into eight sub-clusters. Each dot represents a single cell. **(C)** tSNE or UMAP plots of 5,140 cells generated from SCLCs. The plots are colored by cell cluster, and the cells are clustered into eight sub-clusters. Each dot represents a single cell. **(D)** A heatmap of the top 10 differentially expressed marker genes in each cluster of hAMSCs. **(E)** A heatmap of the top 10 differentially expressed marker genes in each cluster of SCLCs. Violin plots depicting the expression of DEGs in clusters of hAMSCs **(F)** and SCLCs **(G)**.

In a previous study, we demonstrated that SCLCs derived from hAMSCs have an even greater ability to regenerate nerves than do Schwann cells (Chen et al., 2019). To investigate the heterogeneity of SCLCs, hAMSCs were first isolated from patients’ abandoned placenta and then induced into SCLCs. Subsequently, scRNA-seq technology was employed to determine the heterogeneity of hAMSCs and SCLCs (Figure 1A). The single-cell data were pre-filtered using the CellRanger pipeline, yielding a total of 6,367 and 5,924 cells from hAMSCs and SCLCs, respectively. There were 132,641 mean reads, 44,760 median UMI counts, and 6,159 median genes per cell for the hAMSCs. The corresponding figures for the SCLCs were 110,319 mean reads, 29,366 median UMI counts, and 5,007 median genes per cell. Following a second filtration process conducted with the Seurat package, there were 6,008 hAMSCs and 5,140 SCLCs that remained.

We visualized single-cell populations of hAMSCs and SCLCs within a total of eight clusters by creating tSNE and UMAP plots to characterize their heterogeneity (Figures 1B,C). Seurat was used to identify the DEGs within each cluster, and the top 10 genes in each cluster were visualized with a heatmap (Figures 1D,E and Supplementary Table S1). Our analysis of the DEGs in each cluster revealed several subpopulations of hAMSCs and SCLCs with specific capacities. For example, several marker genes of cell growth, such as CCNB1, RRM2, and CENPF, were expressed at high levels in clusters 3 and 4 of the hAMSCs and cluster 7 of the SCLCs, suggesting that these three clusters possessed a stronger proliferation ability compared with other clusters (Figures 1F,G and Supplementary Table S1). Moreover, ECM components, notably DCN, ACTA2, and COL1A2, were expressed at high levels in clusters 0 and 1 of the hAMSCs and in clusters 3 and 4 of the SCLCs (Figures 1F,G and Supplementary Table S1). Several factors associated with tissue regeneration, for example, PPAPA, TIMP1, CTGF, SQSTM1, MMP1, and TIMP3, were also highly expressed in the clusters of hAMSCs and SCLCs. Furthermore, DEGs, such as CD14, CXCL14, and IL24, which are associated with inflammatory processes, were only highly expressed in SCLC clusters (Figures 1F,G and Supplementary Table S1).

A comparison of the bulk RNA-seq and scRNA-seq revealed that whereas the former evidenced 980 up-regulated genes and 682 down-regulated genes in SCLCs, the latter evidenced just 355 up-regulated genes and 389 down-regulated genes in SCLCs (Supplementary Figures 2A,B and Supplementary Table 2). As shown in Supplementary Figure 2C, there were 115 up-regulated genes and 112 down-regulated genes in the overlap between the bulk RNA-seq and the scRNA-seq. The results of the GO and KEGG enrichment analysis indicated that GO terms associated with development, ECM, and angiogenesis, such as “urogenital system development,” “kidney development,” “extracellular matrix organization,” “ECM-receptor interaction,” “blood vessel morphogenesis,” and “angiogenesis” were enriched in the SCLCs (Supplementary Figure 2D). Moreover, the genes involved in these pathways reportedly promote nerve regeneration (Kubiak et al., 2020).

### Cell Cycle Status of hAMSCs and SCLCs

**FIGURE 2:**
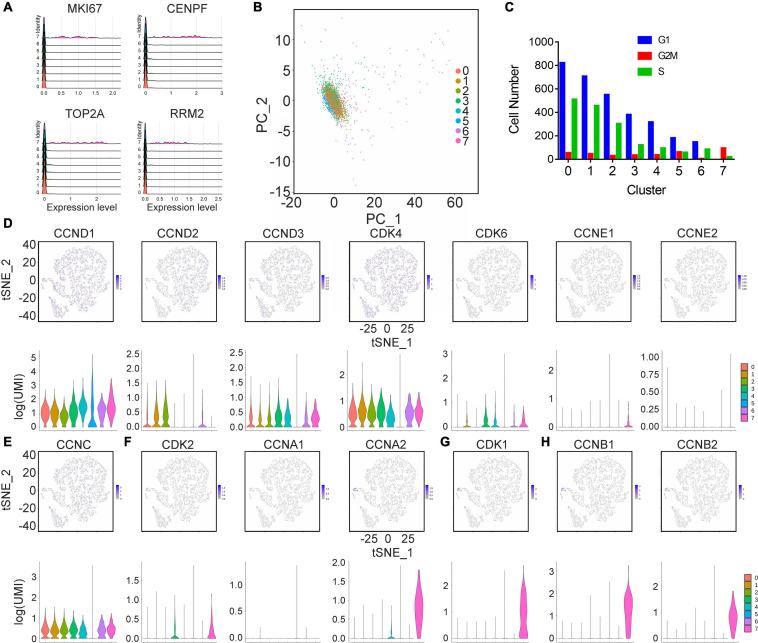
Cell cycle analysis of cells generated from hAMSCs. **(A)** Ridges plot depicting the expression of CLSPN, MKI67, RRM2, and TYMS. **(B)** A PCA plot depicting the cell cycle state of cells from each cluster. **(C)** A bar plot showing the number of cells in different cell cycle states within each cluster. tSNE and violin plots showing the expression of cyclin or cyclin-dependent genes in the following phases: G1 **(D)**, G0 **(E)**, S **(F)**, M **(G)**, and G2 **(H)**.

To characterize the heterogeneity of hAMSCs and SCLCs further, we evaluated the cell cycle status of single cells in hAMSCs and SCLCs. First, cell proliferation markers (CLSPN, MKI67, RRM2, CENPF, TOP2A, and TYMS) were used to determine the proliferation ability of each single cell within each cluster. Ridgeline plots showed that these genes were highly expressed in clusters 3 and 4 of hAMSCs and cluster 7 of SCLCs (Supplementary Figure 3A and Figure 2A), whereas the single cells in other clusters evidenced limited expression of these proliferation markers, thus endorsing the above results (Figures 1F,G). The PCA plot of cell distribution within each cluster during phases of the cell cycle showed that hAMSCs have more proliferating cells compared with SCLCs (Supplementary Figure 3B and Figure 2B). After calculating the number of cells in each cell cycle phase for each cluster, we found that most single cells remained in the G1/S phase. Only single cells in clusters 3 and 4 of the hAMSCs and cluster 7 of the SCLCs were in the G2/M phase (Supplementary Figure 3C and Figure 2C). Lastly, we evaluated the expression of cyclin or cyclin-dependent genes in the following phases: G1 (Supplementary Figure 3D and Figure 2D), G0 (Supplementary Figure 3E and Figure 2E), S (Supplementary Figure 3F and Figure 2F), M (Supplementary Figure 3G and Figure 2G) and G2 (Supplementary Figure 3H and Figure 2H). Our finding that the marker genes for the G2/M phase were highly expressed in clusters 3 and 4 of the hAMSCs, and cluster 7 of the SCLCs, confirmed the higher proliferation capacities of single cells within these three clusters compared with the proliferation capacities of single cells within the other clusters (Supplementary Figures 3D–H and Figures 2D–H).

### Identifying the Transition From hAMSCs to SCLCs Through Pseudotime Analysis

To investigate the mechanism of transformation of hAMSCs into SCLCs, we reanalyzed the merged single-cell populations of hAMSCs plus SCLCs. Single-cell populations of hAMSCs plus SCLCs, aggregated into nine clusters, were visualized using tSNE and UMAP plots (Supplementary Figure 4A). We subsequently evaluated the proportions of each cluster within the population of hAMSCs plus SCLCs. More than 50% of single cells in clusters 0, 4, 7, and 8 were from SCLCs, with cluster 0 being the dominant population among the SCLCs (Supplementary Figure 4B). In clusters 1, 2, 3, 5, and 6, more than 50% of single cells were from hAMSCs, with cluster 1 being the dominant population among the hAMSCs (Supplementary Figure 4B). The differentially expressed genes in each cluster were identified using Seurat, and the top four highly expressed genes in each cluster were visualized with a heatmap (Supplementary Figure 4B).

**FIGURE 3:**
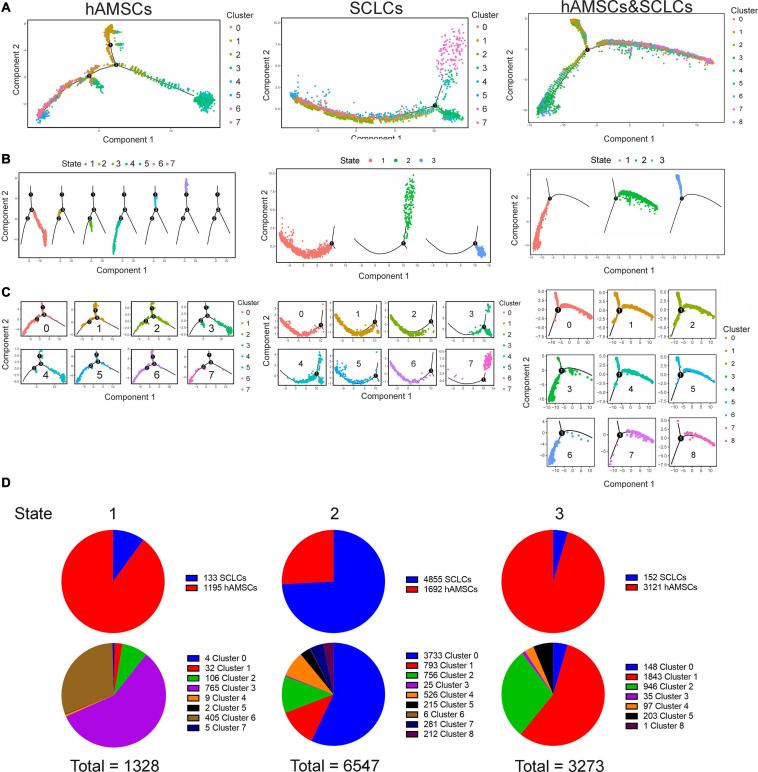
Single-cell trajectories showing the transition from hAMSCs to SCLCs. **(A)** Single-cell trajectories of cells constructed with Monocle, an R package, based on gene expression. Cells are colored by cluster. **(B)** Single-cell trajectories of cells in each state according to gene expression. Cells are colored by state. **(C)** Single-cell trajectories of cells within each cluster according to gene expression. Cells are colored by cluster. **(D)** Pie charts showing the detailed proportion of states in the hAMSC plus SCLC population.

Trajectory analysis of scRNA-seq data was performed to examine the relationship between hAMSCs and SCLCs. According to Pseudotime analysis, single cells within each cluster are grouped into different branches and states based on DEGs. We performed DEG-based Pseudotime analysis on each cluster of hAMSCs, SCLCs, and hAMSCs plus SCLCs (Figure 3A). Three states were identified in the SCLC and hAMSC plus SCLC populations, and seven states were identified in the hAMSC population (Figure 3B), indicating a higher level of variability of single cells in the hAMSC population compared with the other populations. We then evaluated the states of single cells in each cluster of hAMSC, SCLC and hAMSC plus SCLC populations, respectively (Figure 3C). We also calculated the number of single cells in each state or each cluster in the hAMSC plus SCLC population (Figure 3D). Single cells in states 1 and 3 were mainly from hAMSC clusters, and single cells in state 2 were mainly from SCLC clusters (Figure 3D), indicating that single cells of hAMSCs in state 1 may transform into single cells of SCLCs in state 2.

### Transcriptomic Profiling of the Subpopulation With Nerve Regeneration Signatures in SCLCs

**FIGURE 4:**
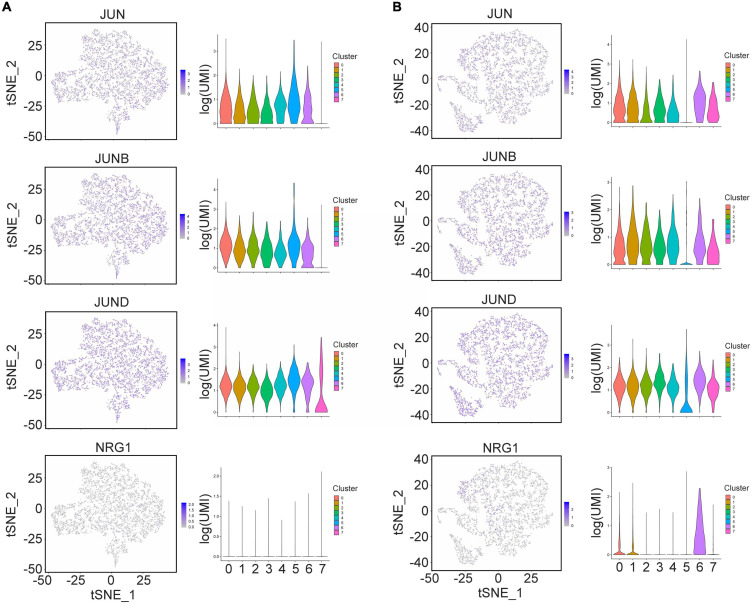
The expression of Schwann cell markers in single hAMSCs and SCLCs. **(A)** The gene expression of Schwann cell markers in hAMSCs visualized in tSNE or violin plots (JUN, JUNB, JUND, and NRG1), respectively. **(B)** The gene expression of Schwann cell markers in SCLCs visualized in tSNE or violin plots (JUN, JUNB, JUND, and NRG1), respectively.

To identify a subpopulation of SCLCs that could play a key role in nerve regeneration, we visualized the gene expression of JUN, JUNB, JUND, and NRG1, which are known regulators of Schwann cells, with tSNE and violin plots (Figure 4). We found that JUN, JUND, and NRG1 were highly expressed in cluster 6 of the SCLCs, but there was little expression of NRG1 in the hAMSC clusters (Figure 4), indicating that cluster 6 of the SCLCs may be the key subpopulation that promotes nerve regeneration.

**FIGURE 5:**
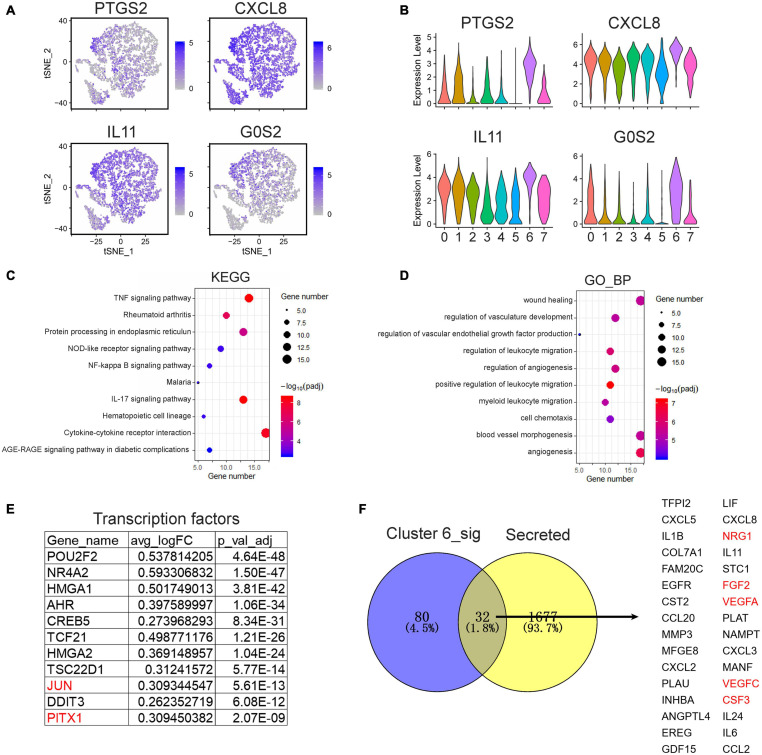
Transcriptomic profiling of cluster 6 SCLCs in the scRNA-seq data. **(A)** tSNE plots and **(B)** violin plots visualizing the expression of PTGS2, CXCL8, IL11, and G0S2. **(C)** enriched KEGG pathways, and **(D)** enriched GO terms of significantly up-regulated genes in cluster 6. **(E)** Top significantly up-regulated transcription factors in cluster 6. **(F)** A Venn diagram showing the significantly up-regulated secreted genes in cluster 6.

The above analysis revealed that single cells in cluster 6 of the SCLCs highly expressed the surface markers of Schwann cells. Profiling of the transcriptomic alteration in cluster 6 of the SCLCs (Figure 5) showed that PTGS2, CXCL8, IL11, and G0S2 were highly expressed in single cells of cluster 6 (Figures 5A,B). We performed enrichment analysis of DEGs in cluster 6. The data indicated the enrichment of KEGG pathways that participate in inflammatory process, such as the TNF signaling pathway, the IL17 signaling pathway, and the NF-kappa B signaling pathway in cluster 6. Further, GO terms associated with tissue repair, such as “angiogenesis,” “wound healing,” “cell chemotaxis,” and “leukocyte migration,” were also enriched in cluster 6 (Figures 5C,D), indicating that single cells in cluster 6 were better able to promote nerve regeneration than those in other clusters. We also examined the highly expressed transcription factors in cluster 6. In addition to JUN, other factors, notably PITX1, which has been shown to be a factor that promotes tissue repair, were also highly expressed in cluster 6, (Figure 5E). A Venn diagram depicting secreted proteins showed that there were 32 overlapping genes between cluster 6 and secreted genes (Uhlen et al., 2019; Figure 5F). Several of these genes, such as VEGFA, FGF2, VEGFC, and CSF3 (Figure 5F) are reportedly growth factors in the tissue repair process. These data suggest that cluster 6 of the SCLCs have transcriptomic signatures with the ability to regenerate nerves.

## Discussion

The regeneration of peripheral nerves after injury poses a challenging problem within clinical practice. Preclinical and clinical studies of stem cell-based therapies for peripheral nerve regeneration have shown that they have potentially promising outcomes (Hopf et al., 2020; Kubiak et al., 2020). Previous studies have shown that SCLCs have an even greater ability to regenerate nerves than do Schwann cells (Jiang et al., 2017). In this study, we evaluated SCLCs at the level of single cells by investigating the heterogeneity of SCLCs via scRNA-seq. Applying clustering with cell cycle status analysis and pseudotime analysis, we identified the heterogeneity of hAMSCs and SCLCs. Moreover, we found that a subpopulation of SCLCs highly express Schwann cell regulators, such as JUN, JUND, and NRG1. Transcriptomic profiling of cluster 6 of the SCLCs indicated that highly expressed genes within this cluster are enriched in pathways that promote nerve regeneration. Our analysis of transcription factors and secreted proteins, notably PITX1, NRG1, and VEGFA, that have been found to promote nerve regeneration (Nocera and Jacob, 2020), are highly expressed within this population.

In recent years, scRNA-seq has been widely used to investigate the heterogeneity of cell types within tumors and different organs, such as the brain (Goldman et al., 2019). Several studies have reported the heterogeneity of cultured mesenchymal stem cells isolated from patients (Barrett et al., 2019; Huang et al., 2019; Khong et al., 2019; Liu et al., 2019; Sun et al., 2020). The heterogeneity of stem cells isolated from umbilical cords, adipose, bone marrow, and Wharton’s jelly have already been investigated. Therefore, in this study, we investigated the heterogeneity of stem cells isolated from hAMSCs. Using a clustering approach and cell cycle analysis, we identified several candidate subpopulations similar to other stem cells, which also showed highly heterogeneity and potential to differentiation. Our pseudotime analysis revealed greater heterogeneity of hAMSCs compared with SCLCs, which suggests that SCLCs’ heterogeneity is limited following their induction. The results of the study demonstrate that after stem cells differentiate into SCLCs, their potential to transform into other cell types may be lost and that only the characteristics of Schwann cells are retained.

Stem cell-based therapies for peripheral nerve regeneration have been widely explored within preclinical and clinical studies (Sullivan et al., 2016; Jiang et al., 2017; Kubiak et al., 2020). Although Schwann cells are key cells involved in the process of nerve regeneration, their clinical application entails limitations (Monje, 2020). Along with other researchers, we have shown that SCLCs induced from mesenchymal stem cells have even better outcomes relating to peripheral nerve generation than do Schwann cells (Jiang et al., 2017; Chen et al., 2019; Hopf et al., 2020). While studies have elucidated several mechanisms behind the promotion of nerve generation by SCLCs, this process is not yet fully understood (Hopf et al., 2020).

In this study, we identified a subpopulation of SCLCs that exhibit nerve regeneration signatures via scRNA-seq. Two groups of genes that are highly expressed within this subpopulation were identified: one is involved in tissue repair and the second in immune control. Transcription factors such as JUN and JUND are critically involved in Schwann cell survival and are activated after PNI (Nocera and Jacob, 2020). NRG1, a key factor in the NRG1/ERBB pathway, is integral for myelination and mediates peripheral nerve development (Modrak et al., 2019). VEGFA not only accelerates the development of the vasculature but it also promotes Schwann cell proliferation, survival, and migration (Namiecińska et al., 2005). Apart from genes promoting tissue repair, those regulating the immune system, such as IL1B, CXCL8, and IL6, which are involved in the TNF and NF-kappa B signaling pathways, are also highly expressed within this subpopulation. In another study, the researchers identified the difference in SCLSs based on proteomic sequencing (Sharma et al., 2017). The authors identified several proteins regulated MSC transdifferentiation which give us more information how MSCs transdifferentiated into SCLCs. These findings suggest that a subpopulation of SCLCs that promotes peripheral nerve regeneration mainly depends on regulating cell survival and immune regulation.

## Conclusion

In conclusion, we applied scRNA-seq in an investigation of the heterogeneity of hAMSCs and SCLCs and identified a subpopulation of SCLCs that exhibit nerve regeneration signatures. However, we only profiled single cells of hAMSCs and SCLCs and did not profile single cells in the intermediate state. Moreover, we did not validate the function of this subpopulation in animal models. In our future work, we are planning to filter out the subpopulation of SCLCs by flow cytometry with surface markers PTGS2/NRG1/EGFR. And we will also validate the nerve regeneration ability of this subpopulation in animal model., Our study provides preliminary elucidation of the role of SCLCs in promoting nerve regeneration at the single cell level, while also providing directions for further research.

## Data Availability Statement

The RNA-seq datasets used for this study are available within the GEO database (GSE161066).

## Ethics Statement

The studies involving human participants were reviewed and approved by Affiliated Hospital of Zunyi Medical University. The patients/participants provided their written informed consent to participate in this study.

## Author Contributions

HL designed and supervised the experiments. ZW, SS, MZ, SX, ST, and KN performed cell culture experiments and single-cell RNA sequencing and analyzed and interpreted the single-cell data. ZW, SS, and HL performed the bioinformatics analysis. ST and KN assisted with the bioinformatics analysis and data interpretation. ZW and HL wrote the manuscript. All authors contributed to the article and approved the submitted version.

## Conflict of Interest

The authors declare that the research was conducted in the absence of any commercial or financial relationships that could be construed as a potential conflict of interest.

## Supplementary Material

Supplementary Method for scRNA-seq analysis.

Supplementary Figure 1 The characterization of hAMSCs.

Supplementary Figure 2 A comparison of bulk RNA-seq and scRNA-seq revealing differences between them.

Supplementary Figure 3 Cell cycle analysis of cells generated from hAMSCs.

Supplementary Figure 4 Single-cell analysis of cells generated from hAMSCs and SCLCs.

Supplementary Table 1 Differentially expressed genes between hAMSCs and SCLCs generated from bulk RNA-seq and scRNA-seq.

Supplementary Table 2 Differentially expressed genes within each cluster of single-cell populations.

## Abbreviations

ATRA: All-trans-retinoic acid
DEGs: Differentially expressed genes
GO: Gene Ontology
hAMSCs: human amniotic mesenchymal stem cells
KEGG: Kyoto Encyclopedia of Genes and Genomes
PBS: phosphate-buffered saline
PNI: Peripheral nerve injury
SCLCs: Schwann cell-like cells
scRNA-seq: single-cell RNA sequencing
UMI: unique molecule identifiers.
