# Supplementary material for: A Subpopulation of Schwann Cell-Like Cells With Nerve Regeneration Signatures Is Identified Through Single-Cell RNA Sequencing
Source: Front Physiol. 2021 May 10;12:637924. doi: 10.3389/fphys.2021.637924 (PMC8171402; doi:10.3389/fphys.2021.637924)
Supplement: Supplementary Figure 1 — The characterization of hAMSCs. [file Data_Sheet_1.docx]

Supplementary Material

# Supplementary Data

## Supplementary method

**Single-Cell RNA Sequencing（scRNA-seq）**

The cell suspension was loaded into Chromium microfluidic chips with 3’ v2 chemistry and barcoded with a 10× Chromium Controller (10X Genomics). RNA from the barcoded cells was subsequently reverse-transcribed and sequencing libraries constructed with reagents from a Chromium Single Cell 3’ v2 reagent kit (10X Genomics) according to the manufacturer’s instructions. Sequencing was performed with NovaSeq according to the manufacturer’s instructions (Illumina).

**Quality control**

We use FastQC to perform basic statistics on the quality of the raw reads. Generally, celltanger count support FASTQ files from raw base call (BCL) files generated by Illumina sequencers as input file.

If cleanreads is indispensable:

Then, raw read sequences produced by the Illumina pipeline in FASTQ format were pre-processed through Trimmomatic software which can be summarized as below:

1. Remove low-quality reads: scan the read with a 4-base wide sliding window, cutting when the average quality per base drops below 10 (SLIDINGWINDOW: 4:10).

(2) Remove trailing low quality or N bases (below quality 3) (TRAILING:3).

(3) Remove adapters: there are two modes to remove the adapter sequence: a. alignment with the adapter sequence, the number of matching bases were greater than 7 and mismatch=2; b.when read1 and read2 overlapping base scoring greater than 30, removed non-overlapping portions (ILLUMINACLIP: adapter.fa: 2: 30: 7).

(4) Drop reads below the 26 bases long.

(5) Discard those reads that cannot form paired. The remaining reads that passed all the filtering steps was counted as clean reads and all subsequent analyses were based on this. At last, we use FastQC to perform basic statistics on the quality of the clean reads.

**Generation and Analysis of Single-Cell Transcriptomes**

Raw reads were demultiplexed and mapped to the reference genome by 10X Genomics Cell Ranger pipeline using default parameters. All downstream single-cell analyses were performed using Cell Ranger and Seurat unless mentioned specifically. In brief, for each gene and each cell barcode (filtered by CellRanger), unique molecule identifiers were counted to construct digital expression matrices. Secondary filtration by Seurat: A gene with expression in more than 3 cells was considered as expressed, and each cell was required to have at least 200 expressed genes. And filter out some of the foreign cells.

In detail:

Cellranger count takes FASTQ files performs alignment, filtering, barcode counting, and UMI counting. It uses the Chromium cellular barcodes to generate feature barcode matrices, determine clusters, and perform gene expression analysis. The count pipeline can take input from multiple sequencing runs on the same GEM well. Add -–nosecondary option to skip secondary analysis of the feature-barcode matrix (dimensionality reduction, clustering and visualization). When doing large studies involving multiple GEM wells, run cellranger count on FASTQ data from each of the GEM wells individually, and then pool the results using cellranger aggr: cellranger aggr aggregates outputs from multiple runs of cellranger count, normalizing those runs to the same sequencing depth and then recomputing the feature-barcode matrices and analysis on the combined data. The aggr pipeline can be used to combine data from multiple samples into an experiment-wide feature-barcode matrix and analysis. Before Secondary Analysis of Gene Expression, we use Seurat Second QC it (as above). Subsequent analysis of cellranger reanalyze and Seurat were all performed basing on this output gene expression matrix.

**Secondary Analysis of Gene Expression by** **cellranger and seurat**

cellranger reanalyze takes feature-barcode matrices produced by cellranger count or cellranger aggr and reruns the dimensionality reduction, clustering, and gene expression algorithms using cellranger default parameter settings. The Seurat package was used to normalize data, dimensionality reduction, clustering, differential expression. we used Seurat alignment method canonical correlation analysis (CCA) for integrated analysis of datasets. For clustering, highly variable genes were selected and the principal components based on those genes used to build a graph, which was segmented with a resolution of 0.6.

# Supplementary Figures and Tables

## Supplementary Figures


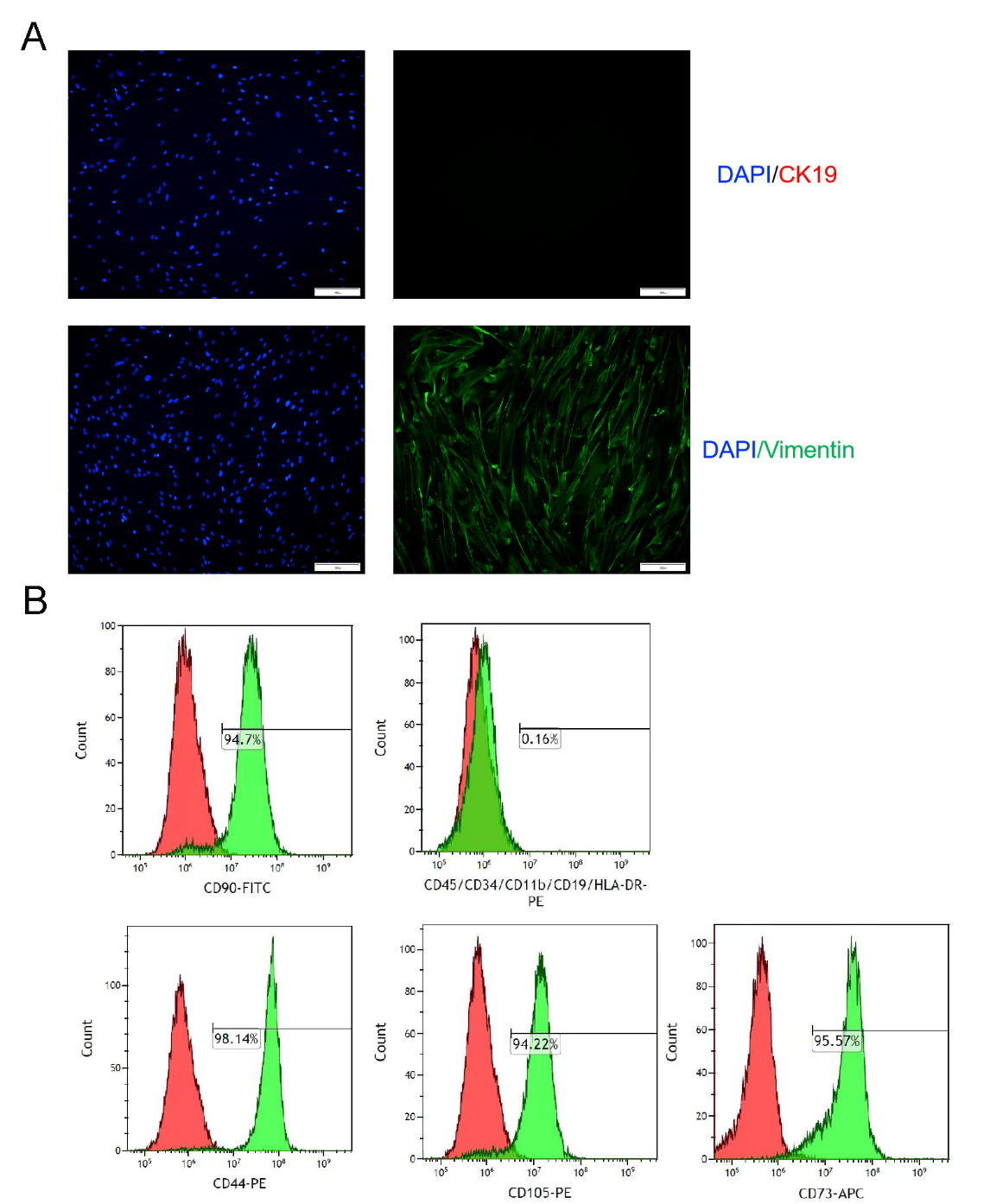


**Supplementary Figure 1.** The characterization of hAMSCs. Immunofluorescence **(A)** and flow cytometry**(B)** were used to characterize hAMSCs. Anti-CK19 and anti-Vimentin were used in Immunoflurescence; CD90, CD44, CD105, CD73 and CD45/CD34/CD11b/CD19 were used for flow cytometry.


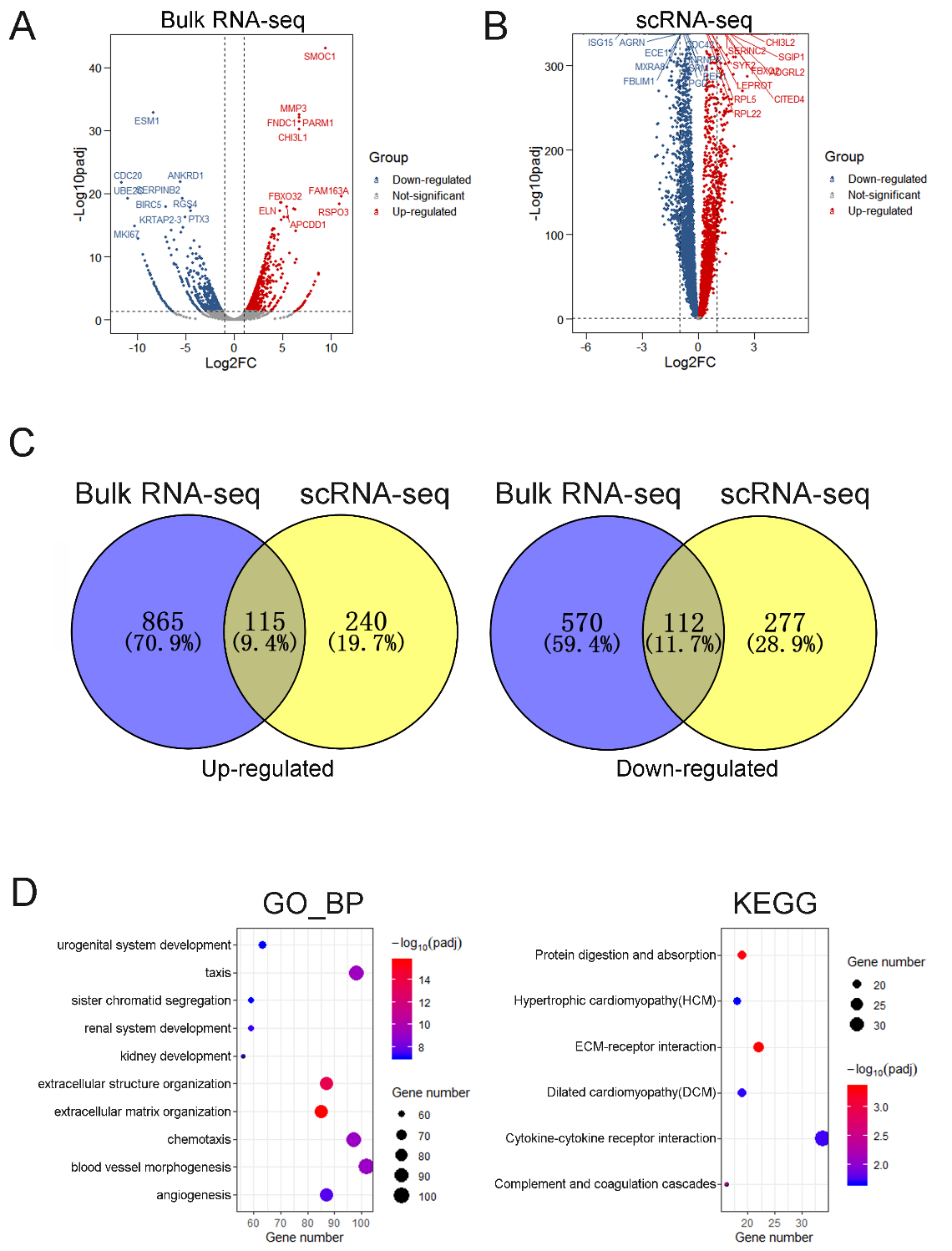


**Supplementary Figure 2.** A comparison of bulk RNA-seq and scRNA-seq revealing differences between them. Volcano plots showing the differentially expressed genes in bulk RNA-seq **(A)** and scRNA-seq **(B)**. **(C)** Venn diagrams showing the differentially expressed genes in the overlap between bulk RNA-seq and scRNA-seq. **(D)** KEGG and GO pathway enrichment analysis of differentially expressed genes in bulk RNA-seq.


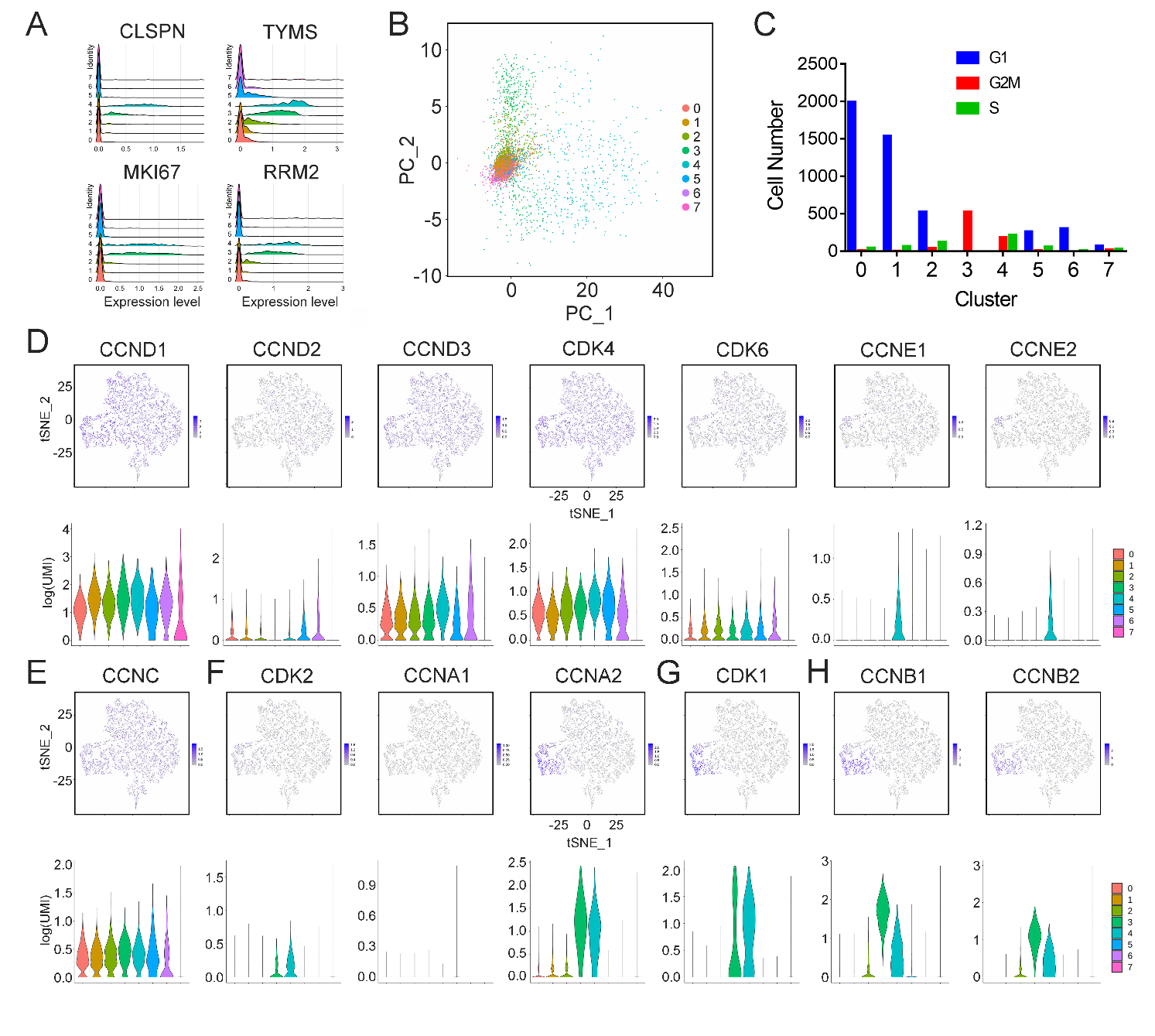


**Supplementary Figure 3.** Cell cycle analysis of cells generated from hAMSCs. **(A)** Ridges plot depicting the expression of CLSPN, MKI67, RRM2, and TYMS. **(B)** A PCA plot depicting the cell cycle state of cells from each cluster. **(C)** A bar plot showing the number of cells in different cell cycle states within each cluster. tSNE and violin plots showing the expression of cyclin or cyclin-dependent genes in the following phases: G1 **(D)**, G0 **(E)**, S **(F)**, M **(G),** and G2 **(H)**.


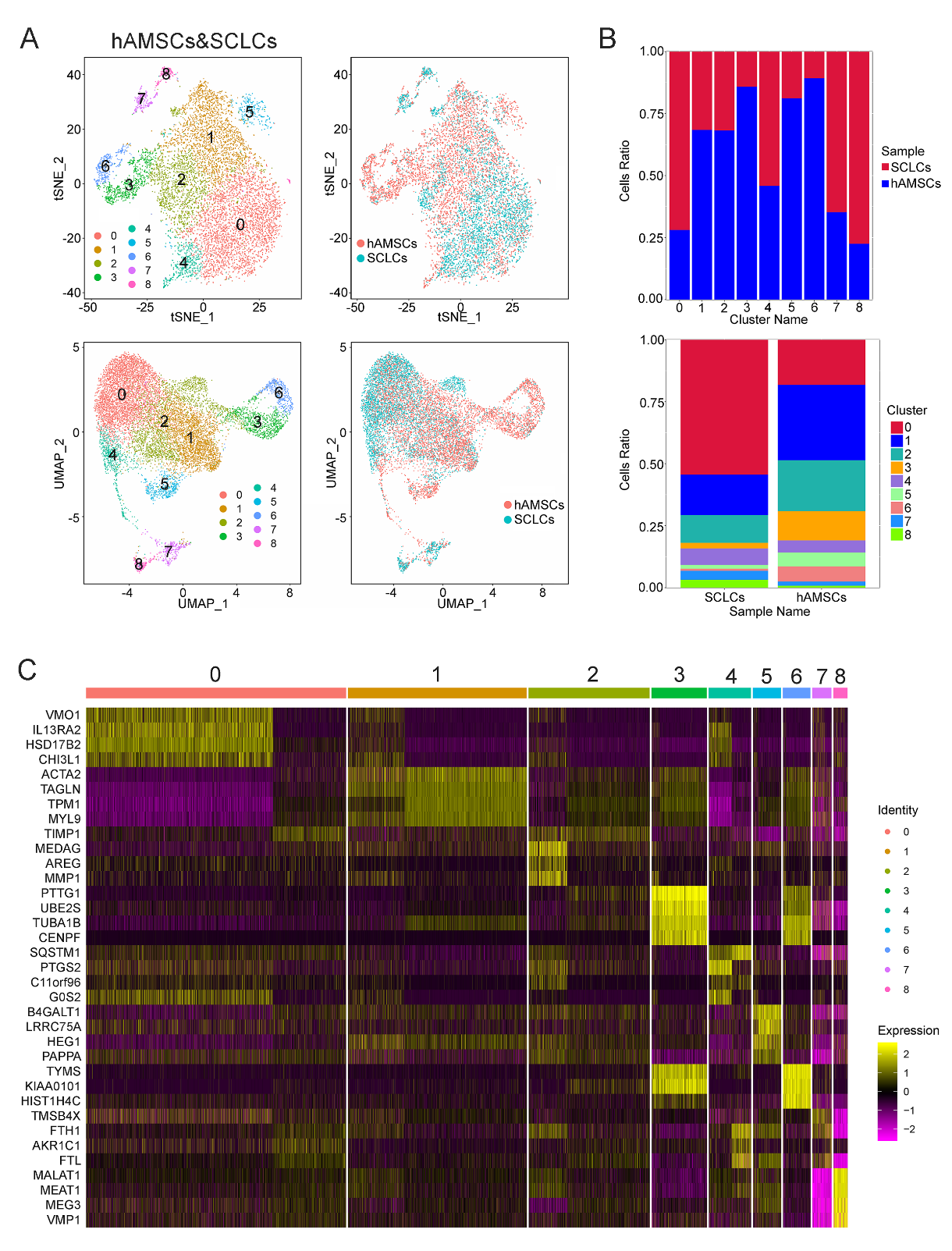


**Supplementary Figure 4.** Single-cell analysis of cells generated from hAMSCs and SCLCs. **(A)** tSNE or UMAP plots depicting a total of 11,148 cells generated from hAMSCs and SCLCs. The plots are colored by cell cluster, and cells are clustered into nine sub-clusters. Each dot represents a single cell. **(B)** A bar plot showing the cell ratio of each sample within different clusters (top) and the cell ratio of clusters in each sample (bottom). **(C)** A heatmap of the top four differentially expressed marker genes within each cluster.
